# Community-based interventions for improving maternal health and for reducing maternal health inequalities in high-income countries: a systematic map of research

**DOI:** 10.1186/s12992-014-0063-y

**Published:** 2015-07-01

**Authors:** Martha Perry, Francisco Becerra, Josephine Kavanagh, Angéline Serre, Emily Vargas, Victor Becerril

**Affiliations:** Health Action Partnership International (HAPI), Unit 31A, Eurolinks Business Centre, 49 Effra Road, Brixton, London, SW2 1BZ UK; Council of Health Research and Development (COHRED), 1-5 Route des Morillons, PO Box 2100, 1211 Geneva 2, Switzerland; Institute of Education, University of London, 20 Bedford Way, WC1H 0AL London, UK; Euroquality, 2, Place de la Bourse, 33000 Bordeaux, France; National Institute of Public Health (Instituto Nacional de Salud Pública), Av. Universidad No. 655 Colonia Santa María Ahuacatitlán, Cerrada Los Pinos y Caminera, C.P. 62100 Cuernavaca, Mor México; National Institute of Public Health (Instituto Nacional de Salud Pública), Av. Universidad No. 655 Colonia Santa María Ahuacatitlán, Cerrada Los Pinos y Caminera, C.P. 62100 Cuernavaca, México

**Keywords:** Maternal health, Inequality, High income country, Systematic review

## Abstract

**Background:**

This review is part of a European Commission project, MASCOT, aimed at reducing maternal and child health inequalities. The purpose was to identify and describe the literature on community-based interventions on maternal health in high-income countries (HIC) and conceptually map the literature according to country focus, topics addressed, nature of the intervention and the intervention provider, and interventions designed to address inequalities in maternal health.

**Methods:**

The research protocol for this review was based on a low-income country (LMIC) systematic review protocol within the MASCOT Project. We searched PubMED and CINAHL databases for literature published between January 2000 and April 2013. OECD countries were used to determine the HIC and different terms were used to refer to community based interventions, defined as those “delivered in community settings or any activities occurring outside of health facilities”.

**Results:**

119 publications were selected for inclusion in this mapping study. 95 (80%) were Randomised Control Trials (RCTs) and 24 (20%) were systematic reviews (SRs). We categorised the study topics according to the main interventions covered: breastfeeding assistance and promotion, preventing and treating post-natal depression, interventions to support and build capacity around parenting and child care, antenatal interventions preparing women for birth, postnatal planning of future births and control trials around changing maternal behaviours. The home was used as the most common setting to implement these interventions and health professionals accounted for the largest group of intervention providers.

**Conclusions:**

This review maps and brings knowledge on the type of studies and topics being addressed in community based interventions around maternal health in HICs. It opens the opportunity for further studies on interventions’ effectiveness and knowledge transfer to LMICs settings.

## Background

Health inequalities are defined as *differences in health status or in the distribution of health determinants between different population groups* [1]. The part of these inequalities attributable to the external environment and conditions outside the control of individuals result in disparities for disease incidence, health outcomes, access to health care, or quality of health care. They are particularly unjust and unfair and therefore referred to as inequities. The WHO Commission on the social determinants of health states that “*the poor health of the poor, the social gradients in health and the marked health inequities between countries are caused by the unequal distribution of power, income, goods and services*” [2] which means they could be avoidable. In the perspective of the Millennium Development Goals, numerous social and economic arguments appeal for a reduction of these inequities.

The “Multilateral Association for Studying health inequalities and enhancing north–south and south-south Cooperation” (MASCOT) [3] recognizes the need of stimulating knowledge transfer and exchange mechanisms among Low and Middle Income Countries (LMIC), as well as with High Income Countries (HIC) for shaping policies, programmes and health actions intended to provide better health and health services. The actions implemented in the framework of MASCOT are understood as a way to reduce inequities preferentially affecting children, adolescents and mothers as the end-result of the strengthened collaborative activities.

A primary objective of MASCOT was to identify the strategies and more particularly the health system interventions that are in place aiming at improving maternal health. As to complement country level work, a systematic review examined evidence of the impact of different supply and demand initiatives on maternal health in LMICs [4], taking a broad approach to health systems and also including community-based interventions, which often provide services and support for marginalised and disadvantaged populations. The preliminary results revealed community-based interventions in the LMIC literature only accounted for 2% of studies. The review was further expanded to include community-based interventions in HICs reinforcing the north–south collaboration and knowledge transfer embedded in MASCOT.

This paper reports the results of a systematic mapping of research, rather than a systematic review. Systematic maps follow the early stages of a systematic review, but do not attempt to critically appraise the literature, nor to extract, synthesise and analyse data. They can be used to: describe a body of literature which is not well understood or difficult to access; identify gaps in the literature; identify topics for systematic reviews; and, make research more easily accessible to policy makers and practitioners, researchers and other users of research.

The objectives of this systematic map were to:

1. systematically identify the literature on community-based interventions on maternal health in HICs; and
2. describe the literature according to concepts such as: country focus, topics addressed, nature of the intervention and the intervention provider, and interventions designed to address inequalities in maternal health.

## Methods

The research protocol for this review was based on a LMIC systematic review protocol [4] within the MASCOT Project. Maternal Health was considered as “the time from conception until two years after childbirth”, thus covering pregnancy, childbirth and the postnatal period. Community-based interventions were defined as those “delivered in community settings or any activities occurring outside of health facilities”. This definition was selected given the size of the review team and number of references to screen, making it difficult to operationalize a more complex definition of community.

We searched PubMED and CINAHL databases for literature published between January 2000 and April 2013. The latter extends one more year than the LMIC review, which search was limited to March 2012. We did not restrict publication type and languages were restricted to those of the MASCOT project country partners (English, French, Spanish and Portuguese).

OECD countries were used to determine the high-income countries [5]. Together, the HIC and LMIC reviews thus cover all countries.

Searches for community-based interventions combined free-text and controlled language terms which describe community interventions for maternal health and we also included specific terms identified in a WHO project on maternal health community interventions. We used a combination of the following terms: “social support” OR “husband” OR “Women’s health groups” OR “Women’s groups” OR “participatory intervention” OR “Lay health worker*” OR “home based” OR “home visit*” OR “Maternity waiting home*” OR “Birth preparedness” OR “Male involvement” OR “Transport scheme*” OR “community scheme*” OR “traditional birth attendant*” OR community OR “community organisation*”OR “community organization*” OR “Social Support” OR (“lay community” OR “lay people” OR “lay person” OR “peer deliver*” OR “peer support”) OR “Community Networks” OR “Community Health Workers” OR “Community-Based Participatory Research” OR “Consumer Participation”.

Following systematic review methodology, publications were selected for full text review if they were either Randomised Control Trials (RCT) or Systematic reviews (SR). The criteria used to determine the eligibility of studies is included in Table 1. Single screening on title and abstract was performed by five reviewers (MP, FB, JK, EV and VB). During screening, references were marked as ‘exclude’ taking a hierarchy approach and marking only the highest applicable item on the list. Included items were marked either ‘include RCT/SR’ or ‘include on TI/AB’, which referred to those studies which were maternal health community-based interventions, but the study designs were not RCTs or SRs.

**Table 1:** **Eligibility criteria of publications**

**Exclusion criteria**	**Inclusion criteria for full text coding**
**1. Published before 2000**	1. Study design was a Randomised Control Trial or systematic review
**2. Not maternal health population (women in pregnancy, childbirth, or within two years postpartum)**	
**3. Not a community-based intervention**	
**4. Not a High-Income country**	
**5. Not research**	

Full text coding of RCTs and SRs was done by three reviewers (MP, FB and AS). First full text articles were checked for eligibility. Then data from the full text was extracted according to a set of generic codes shown in Table 2. These generic codes were developed by the LMIC systematic review team and this review used the same to ensure the studies from HIC literature could be classified similarly for comparison. In addition, a set of specific codes were added for HIC literature on the type of community-setting where the intervention was delivered and on who the intervention provider was. Besides health professionals, providers were either peers, defined as “women who have themselves had children or have the same socioeconomic background, ethnicity, or locality as the women they are supporting” and community volunteers, who are different from peers in that they were not mothers or women necessarily.

**Table 2:** **Generic codes applied to full text**

1. Country(ies) where study done	8. Intervention topic.
2. Country(ies) of first author	9. Period targeted by intervention: Pregnancy (which includes abortion and miscarriage); Intrapartum; Postpartum.
3. Paper targeted at or delivered to specific PROGRESS-Plus group or disadvantaged group as defined by PROGRESS-Plus. These categories are: Place of Residence (rural women for example), Race/Ethnicity, Occupation, Religion, Education, Socioeconomic Status, and Social Capital, and Plus represents additional categories such as Age, Disability and Sexual Orientation.	10. Data collected: maternal health; child health; cost/health economics; service utilisation
4. Paper addresses WHO health promotion topics. This includes health promotion activities and health education activities within the community, and for the community, including that which occurs in health service settings.	11. Funding
5. Research question(s) study might answer: Health systems; Community settings; Tracer conditions/single clinical interventions; Tracer conditions/other interventions; Health service utilisation/non-intervention research; Health promotion; Other.	12. HIC Codes
6. Study design: RCT or SR	a. Intervention delivery: home; telephone; peer delivered; other
7. Intervention recipient: women; family; male partner; traditional birth attendant; community health worker; midwife/nurse; other mid-level provider; doctor/obstetrician; community; manager(s); policy maker(s); system; rural setting; urban setting; other.	b. Intervention provider: health professional; peer; community volunteer; other

Because one of the principal objectives of the MASCOT project is to share knowledge and build capacity to reduce maternal and child health inequities, we also coded included articles on whether the intervention targeted disadvantaged populations. We define disadvantage through the acronym PROGRESS-Plus (Place of Residence, Race/Ethnicity, Occupation, Gender, Religion, Education, Socioeconomic Status, and Social Capital, and Plus represents additional categories such as Age, Disability, and Sexual Orientation), used by the Campbell and Cochrane Equity methods Group and the Cochrane Public Health Review Group [4].

**Figure 1:**
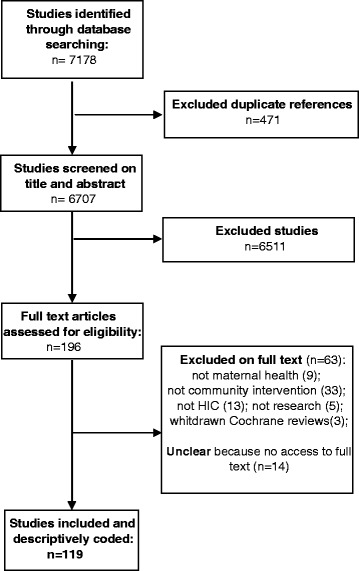
**Flow of study selection.**

A total of 7178 documents were obtained from the literature search, from which 119 publications were selected for further analysis following the review process shown in the study flow diagram (Figure 1). The list of publications is included in Appendix.

## Results

Of the included publications, 95 (80%) were RCTs and 24 (20%) SRs, which characteristics and country of origin are presented in Table 3. Because of the definition used for this work of community-based interventions, studies were focussed on prevention and health promotion for mothers, infants and families. Breastfeeding assistance and promotion (n = 27; 22.7%), preventing and treating post-natal depression (n = 30; 25.2%) or interventions to support and build capacity around parenting and child care (n = 27; 22.6%) accounted for about three thirds of studies (n = 84; 70.5%). The rest were primarily on antenatal interventions preparing women for birth (n = 9; 7.6%), postnatal planning of future births (n = 7; 5.8%) and control trials around changing maternal behaviours, including nutrition and physical activity promotion (n = 5; 4.2%), smoking cessation (n = 5; 4.2%) and drug use prevention (n = 4; 3.7%).

**Table 3:** **Results of conceptual mapping of included references**

**Coding categories**	**All (n = 119)**	**Randomised control trials (n = 95)**	**Systematic reviews (n = 24)**
Countries			
Australia		13 (13.7%)	12 (50.0%)*
Canada		14 (14.7%)	14 (58.3%)
United Kingdom		16 (16.8%)	17 (70.8%)
United States		42 (44.2%)	18 (75.0%)
Other HIC		10 (10.6%)	15 (62.5%)
Topic			
Birth preparedness	9 (7.6%)	8 (8.4%)	1 (4.2%)
Breastfeeding	27 (22.7%)	16 (16.8%)	11 (45.8%)
Drug use	4 (3.7%)	4 (4.2%)	0 (0.0%)
Family planning	7 (5.8%)	7 (7.4%)	0 (0.0%)
Nutrition and/or physical activity	5 (4.2%)	5 (5.3%)	0 (0.0%)
Parenting and child care	27 (22.7%)	24 (25.3%)	3 (12.5%)
Post-natal depression	30 (25.2%)	24 (25.3%)	6 (25.0%)
Smoking cessation	5 (4.2%)	4 (4.2%)	1 (4.2%)
Other	5 (4.2%)	3 (3.1%)	2 (8.3%)
Intervention delivery			
Home only	44 (37.0%)	40 (42.1%)	4 (16.7%)
Peer only	6 (5.0%)	4 (4.2%)	2 (8.3%)
Telephone only	7 (5.9%)	6 (6.3%)	1 (4.2%)
Combined community settings	32 (26.9%)	21 (22.1%)	11 (45.8%)
Combined community and health facility	30 (25.2%)	24 (25.3%)	6 (25%)
Intervention provider			
Community volunteer	5 (4.2%)	4 (4.2%)	1 (4.2%)
Peer	23 (19.3%)	21 (22.1%)	2 (8.3%)
Health Professional	61 (51.3%)	52 (54.8%)	9 (37.5%)
Combined health worker and peer and/or community volunteer	26 (21.8%)	14 (14.7%)	12 (50.0%)
Other	4 (3.4%)	4 (4.2%)	0 (0.0%)
Population targeted			
PROGRESS Plus group	39 (32.8%)	35 (36.8%)	4 (16.7%)
Universal	80 (67.2%)	60 (63.2%)	20 (83.3%)

*The percentage represents the number of times that there are one or more studies from the country in the 24 systematic reviews included in this scoping study.

Service delivery mechanisms were generally interventions at the home (n = 44; 37.0%) and through telephone support (n = 7; 5.9%) or peer support groups (n = 6; 5.0%), and a little over one quarter of studies (n = 32; 26.9%) combined one or more of these; for instance a breastfeeding home visitation programme with additional telephone support. We also included studies which compared mainstream hospital or clinical care with enhanced care outside of the health setting, as well as those which combined health facility care with one or more of the community-setting support (n = 30; 25.2%). Over half of these interventions (n = 61; 51.3%) were provided by health professionals, which included nurses, doctors, midwives and also research groups from health-related disciplines. Interventions were provided by peers in 19.3% (n = 23) of studies and 4.2% (n = 5) by community volunteers. Both health and non-health intervention providers accounted for 21.8% (n = 26) of included studies.

The outcomes presented in the studies (summarised in Table 4) were mainly maternal health (n = 111; 93.3%) and child health (n = 51; 42.9%). The other two were related to costs (n = 14; 11.8%), in particular analysing any benefits or reductions in healthcare costs from community-based support, and service utilisation (n = 17; 14.3%), which was related both to interventions promoting the use of antenatal and post-natal services and service use post-intervention.

**Table 4:** **Type of data collected in included references**

**Data collected**	**Number of articles**
Maternal health outcomes	111 (93.3%)
Child health outcomes	51 (42.9%)
Service Utilisation	17 (14.3%)
Cost/Health economics	14 (11.8%)

In relation to the PROGRESS-Plus classification, 39 studies (32.8%) included interventions which were targeted to specific population groups. As presented in Table 5, they were primarily women in low socioeconomic strata (n = 21; 17.6%), adolescents (n = 14; 11.8%), Black and ethnic minorities (n = 10; 8.4%) and others (n = 6; 5.0%) referred to indicators such as living in urban areas, being a single parent or born overseas.

**Table 5:** **PROGRESS Plus Population targeted in included references**

**PROGRESS-Plus group**	**Number of articles**
Low socio-economic status	21 (17.6%)
Adolescents	14 (11.8%)
Black and ethnic minorities	10 (8.4%)
Other	6 (5.0%)

The largest mentioned source of funding was governmental (n = 80; 67.2%) followed by not-for-profit organisations (n = 21; 17.6%), academic institutions (n = 11; 8.7%) and private sector (n = 4; 3.3%) (refer to Table 6). Only one systematic review [6] had international agency funding from the World Health Organization and European Commission.

**Table 6:** **Sources of funding for community-based interventions in HIC**

**Funding source**	**Number of articles**
Government agency	80 (67.2%)
No funding acknowledged	26 (21.8%)
Not-for-profit organisation	21 (17.6%)
Academic institution	11 (8.7%)
Private sector organisation	4 (3.3%)
International organisation	1 (0.8%)

## Discussion

In the literature on community-based interventions in maternal health from HICs in recent years, post-natal depression, breastfeeding and parenting are the main focus of interventions. Together, these topics represent 71% of all studies. According to the Global burden of Disease Heatmap, depressive disorder which amounts to 2.55% of the global burden of disease [7] is the 11th cause globally, being higher in Latin American and Caribbean countries than in HICs, but amongst the 10th place in some parts of the region, while in Africa it ranges between the 10th (Southern Sub-Sahara) and the 19th place (Western Sub-Saharan Africa) [8] Furthermore, the World Health Organization reports the burden of depression is 50% higher in women than men, and it estimates between 1 and 2 out of 10 mothers will experience post-natal depression to various degrees, which can limit their capacity to care for their children [9].

It is interesting to find that most of the RCTs were either comparing community-based interventions to health care facility services or providing a combination of both. This can be explained by the non-clinical nature of the studies’ topics, which may not necessarily require the use or attendance at a health facility. Furthermore, arguments and evidence around cost-saving and cost-effectiveness to both the health sector and the mothers with the use of community settings can also be made. Mothers’ homes were a common setting used to provide support outside of mainstream health service provision. Home visiting interventions are often deigned to target women who are socially excluded, living in poverty or undereducated [10] and in our findings approximately 85% (n = 33) of the studies on disadvantaged women (n = 39) included home support.

The outcomes of maternal health programmes can also be assessed in terms of the background of the intervention provider. Half of the studies used health workers followed by combination of health worker and other lay type of supporter. The third most used intervention provider were peers based on an assumption that they can provide support for mothers and share commonalities which enhance confidence and trust [11,12]. Further evaluation of included studies can identify the effect of different types of providers, but this analysis was outside of the aim of this paper.

The scope of the targeted population by the HIC studies is mainly universal for both the RCT and SR studies. Of the PROGRESS Plus categories, adolescents, black and ethnic minorities and low socio-economic status are the main populations targeted. Adolescent pregnancy is high worldwide and one of the main causes of maternal death [13]. Teenagers tend to have more complications than women who have reached an adult age [14]. The problem is greater in the United States and Canada than in Europe and Japan, and is not only due to high pregnancy rates in minority groups in the United States, but also access to information and education on sexual health which play a positive role in preventing adolescent pregnancy [15].

Funding for these studies comes mainly from governments and not-for-profit organizations. This may be a reflection of the interest the issues being investigated and trying to find evidence-based models to respond to some of the current problems HIC have around maternal health. Because of the focus of this study on those interventions outside health settings, treating post-natal depression is the only medical condition addressed. This does not mean that women in HICs do not experience other type of complications or conditions during pregnancy, childbirth and post-partum. Maternal mortality remains a global problem. A report comparing global Maternal Mortality Ratios (MMR) - the number of women dying for every 100,000 live births- found that in 2008 mothers in the United States died at a higher rate (16.7) than in most other high-income countries, they were followed by France (10.0), Denmark (9.4) and the United Kingdom (8.2); while Australia (5.1), Sweden (4.6) and Italy (3.9) had the lowest MMR [16].

## Conclusions

The review aimed to produce a descriptive map of research on community based interventions, but did not look into the further implementation of the proven strategies as to make them full-status programmes, nor did it look into the analysis of success rates and meta-analysis.

This is the only systematic map attempting to cover community-based interventions around maternal health in HIC that the authors are aware of. This systematic map is useful as it brings knowledge on the type of studies and topics being addressed, opening the opportunity for further studies or a full systematic review assessing the effectiveness of a range of community based interventions. One of the principal objectives of the MASCOT project is to share knowledge and build capacity to reduce maternal and child health inequities,. The inclusion of community based interventions in HIC was considered to be of particular relevance due to increased interest in these types of interventions in LMICs, an interest which has yet to be matched with relevant research. Identifying and describing what is being done in HICs is a useful starting point to open learning perspectives between LMICs and HICs. HIC standards and perceptions might also play a role in the definitions of the studies and the people involved in delivering the services and support. The role of information channels and publicity might also create awareness on the selected topics for the studies by the researchers, as previously noted, post-natal depression was the key topic on studies. Evidence is central to inform policy and practice. Further research is needed to analyse and compare the effectiveness of the interventions mapped in this study to make recommendations to policy makers and practitioners. Moreover, it would be useful to include literature on community-based interventions in LMIC identified through the MASCOT project to better understand differences between settings and be able to inform other priority areas related to maternal wellbeing and parenting, in addition to preventing birth complications and providing safe deliveries.

## Appendix

1. Ahmed AH, Sands LP. Effect of pre- and postdischarge interventions on breastfeeding outcomes and weight gain among premature infants. Journal of obstetric, gynecologic, and neonatal nursing: JOGNN/NAACOG. 2010;39(1):53–63.

2. Ammerman RT, Putnam FW, Altaye M, Teeters AR, Stevens J, Van Ginkel JB. Treatment of depressed mothers in home visiting: Impact on psychological distress and social functioning. Child abuse & neglect. 2013.

3. Armstrong K, Edwards H. The effects of exercise and social support on mothers reporting depressive symptoms: a pilot randomized controlled trial. International journal of mental health nursing. 2003;12(2):130–8.

4. Armstrong K, Edwards H. The effectiveness of a pram-walking exercise programme in reducing depressive symptomatology for postnatal women. International journal of nursing practice. 2004;10(4):177–94.

5. Armstrong KL, Fraser JA, Dadds MR, Morris J. Promoting secure attachment, maternal mood and child health in a vulnerable population: a randomized controlled trial. Journal of paediatrics and child health. 2000;36(6):555–62.

6. Au F, Shiell A, van der Pol M, Johnston DW, Tough S. Does supplementary prenatal nursing and home visitation reduce healthcare costs in the year after childbirth? Journal of advanced nursing. 2006;56(6):657–68.

7. Barlow A, Varipatis-Baker E, Speakman K, Ginsburg G, Friberg I, Goklish N, et al. Home-visiting intervention to improve child care among American Indian adolescent mothers: a randomized trial. Archives of pediatrics & adolescent medicine. 2006;160(11):1101–7.

8. Barlow J, Davis H, McIntosh E, Jarrett P, Mockford C, Stewart-Brown S. Role of home visiting in improving parenting and health in families at risk of abuse and neglect: results of a multicentre randomised controlled trial and economic evaluation. Archives of disease in childhood. 2007;92(3):229–33.

9. Barnes J, Senior R, MacPherson K. The utility of volunteer home-visiting support to prevent maternal depression in the first year of life. Child: care, health and development. 2009;35(6):807–16.

10. Barnet B, Liu J, DeVoe M, Alperovitz-Bichell K, Duggan AK. Home visiting for adolescent mothers: effects on parenting, maternal life course, and primary care linkage. Annals of family medicine. 2007;5(3):224–32.

11. Barnet B, Liu J, DeVoe M, Duggan AK, Gold MA, Pecukonis E. Motivational intervention to reduce rapid subsequent births to adolescent mothers: a community-based randomized trial. Annals of family medicine. 2009;7(5):436–45.

12. Barnet B, Rapp T, DeVoe M, Mullins CD. Cost-effectiveness of a motivational intervention to reduce rapid repeated childbearing in high-risk adolescent mothers: a rebirth of economic and policy considerations. Archives of pediatrics & adolescent medicine. 2010;164(4):370–6.

13. Bartu A, Sharp J, Ludlow J, Doherty DA. Postnatal home visiting for illicit drug-using mothers and their infants: a randomised controlled trial. The Australian & New Zealand journal of obstetrics & gynaecology. 2006;46(5):419–26.

14. Beeber LS, Holditch-Davis D, Belyea MJ, Funk SG, Canuso R. In-home intervention for depressive symptoms with low-income mothers of infants and toddlers in the United States. Health care for women international. 2004;25(6):561–80.

15. Boulvain M, Perneger TV, Othenin-Girard V, Petrou S, Berner M, Irion O. Home-based versus hospital-based postnatal care: a randomised trial. BJOG: an international journal of obstetrics and gynaecology. 2004;111(8):807–13.

16. Brooten D, Youngblut J, Blais K, Donahue D, Cruz I, Lightbourne M. APN-physician collaboration in caring for women with high-risk pregnancies. Journal of nursing scholarship: an official publication of Sigma Theta Tau International Honor Society of Nursing/Sigma Theta Tau. 2005;37(2):178–84.

17. Brooten D, Youngblut JM, Brown L, Finkler SA, Neff DF, Madigan E. A randomized trial of nurse specialist home care for women with high-risk pregnancies: outcomes and costs. The American journal of managed care. 2001;7(8):793–803.

18. Chapman DJ, Damio G, Young S, Perez-Escamilla R. Effectiveness of breastfeeding peer counseling in a low-income, predominantly Latina population: a randomized controlled trial. Archives of pediatrics & adolescent medicine. 2004;158(9):897–902.

19. Chapman DJ, Morel K, Anderson AK, Damio G, Perez-Escamilla R. Breastfeeding peer counseling: from efficacy through scale-up. Journal of human lactation: official journal of International Lactation Consultant Association. 2010;26(3):314–26.

20. Cheng S, Kondo N, Aoki Y, Kitamura Y, Takeda Y, Yamagata Z. The effectiveness of early intervention and the factors related to child behavioural problems at age 2: a randomized controlled trial. Early human development. 2007;83(10):683–91.

21. Daley A, Jolly K, MacArthur C. The effectiveness of exercise in the management of post-natal depression: systematic review and meta-analysis. Family practice. 2009;26(2):154–62.

22. Dennis CL. Breastfeeding peer support: maternal and volunteer perceptions from a randomized controlled trial. Birth (Berkeley, Calif). 2002;29(3):169–76.

23. Dennis CL. Treatment of postpartum depression, part 2: a critical review of nonbiological interventions. The Journal of clinical psychiatry. 2004;65(9):1252–65.

24. Dennis CL. Postpartum depression peer support: maternal perceptions from a randomized controlled trial. International journal of nursing studies. 2010;47(5):560–8.

25. Dennis CL, Dowswell T. Psychosocial and psychological interventions for preventing postpartum depression. Cochrane database of systematic reviews (Online). 2013;2:CD001134.

26. Dennis CL, Hodnett E, Gallop R, Chalmers B. The effect of peer support on breast-feeding duration among primiparous women: a randomized controlled trial. CMAJ: Canadian Medical Association journal = journal de l’Association medicale canadienne. 2002;166(1):21–8.

27. Dennis CL, Hodnett E, Kenton L, Weston J, Zupancic J, Stewart DE, et al. Effect of peer support on prevention of postnatal depression among high risk women: multisite randomised controlled trial. BMJ (Clinical research ed). 2009;338:a3064.

28. Dennis CL, Kingston D. A systematic review of telephone support for women during pregnancy and the early postpartum period. Journal of obstetric, gynecologic, and neonatal nursing: JOGNN/NAACOG. 2008;37(3):301–14.

29. Donatelle RJ, Prows SL, Champeau D, Hudson D. Randomised controlled trial using social support and financial incentives for high risk pregnant smokers: significant other supporter (SOS) program. Tobacco control. 2000;9 Suppl 3:III67-9.

30. Dowswell T, Renfrew MJ, Gregson B, Hewison J. A review of the literature on women’s views on their maternity care in the community in the UK. Midwifery. 2001;17(3):194–202.

31. Dowswell T, Renfrew MJ, Hewison J, Gregson BA. A review of the literature on the midwife and community-based maternity care. Midwifery. 2001;17(2):93–101.

32. Dritsa M, Da Costa D, Dupuis G, Lowensteyn I, Khalife S. Effects of a home-based exercise intervention on fatigue in postpartum depressed women: results of a randomized controlled trial. Annals of behavioral medicine: a publication of the Society of Behavioral Medicine. 2008;35(2):179–87.

33. Dritsa M, Dupuis G, Lowensteyn I, Da Costa D. Effects of home-based exercise on fatigue in postpartum depressed women: who is more likely to benefit and why? Journal of psychosomatic research. 2009;67(2):159–63.

34. D’Souza L, Garcia J. Improving services for disadvantaged childbearing women. Child: care, health and development. 2004;30(6):599–611.

35. El-Kamary SS, Higman SM, Fuddy L, McFarlane E, Sia C, Duggan AK. Hawaii’s Healthy Start home visiting program: determinants and impact of rapid repeat birth. Pediatrics. 2004;114(3):e317-NaN.

36. Escobar GJ, Braveman PA, Ackerson L, Odouli R, Coleman-Phox K, Capra AM, et al. A randomized comparison of home visits and hospital-based group follow-up visits after early postpartum discharge. Pediatrics. 2001;108(3):719–27.

37. Fergusson DM, Grant H, Horwood LJ, Ridder EM. Randomized trial of the Early Start program of home visitation: parent and family outcomes. Pediatrics. 2006;117(3):781–6.

38. Gagnon AJ, Dougherty G, Jimenez V, Leduc N. Randomized trial of postpartum care after hospital discharge. Pediatrics. 2002;109(6):1074–80.

39. Gagnon AJ, Sandall J. Individual or group antenatal education for childbirth or parenthood, or both. Cochrane database of systematic reviews (Online). 2007(3):CD002869.

40. Gedde-Dahl M, Fors EA. Impact of self-administered relaxation and guided imagery techniques during final trimester and birth. Complementary therapies in clinical practice. 2012;18(1):60–5.

41. Ginsburg GS, Barlow A, Goklish N, Hastings R, Baker EV, Mullany B, et al. Postpartum Depression Prevention for Reservation-Based American Indians: Results from a Pilot Randomized Controlled Trial. Child & youth care forum. 2012;41(3):229–45.

42. Goulet C, Gevry H, Gauthier RJ, Lepage L, Fraser W, Aita M. A controlled clinical trial of home care management versus hospital care management for preterm labour. International journal of nursing studies. 2001;38(3):259–69.

43. Graffy J, Taylor J, Williams A, Eldridge S. Randomised controlled trial of support from volunteer counsellors for mothers considering breast feeding. BMJ (Clinical research ed). 2004;328(7430):26.

44. Grant TM, Ernst CC, Streissguth A, Stark K. Preventing alcohol and drug exposed births in Washington state: intervention findings from three parent–child assistance program sites. The American journal of drug and alcohol abuse. 2005;31(3):471–90.

45. Hannan J. APN telephone follow up to low-income first time mothers. Journal of clinical nursing. 2013;22(1–2):262–70.

46. Hayes BA, Muller R, Bradley BS. Perinatal depression: a randomized controlled trial of an antenatal education intervention for primiparas. Birth (Berkeley, Calif). 2001;28(1):28–35.

47. Homer CS, Davis GK, Brodie PM. What do women feel about community-based antenatal care? Australian and New Zealand journal of public health. 2000;24(6):590–5.

48. Homer CS, Davis GK, Brodie PM, Sheehan A, Barclay LM, Wills J, et al. Collaboration in maternity care: a randomised controlled trial comparing community-based continuity of care with standard hospital care. BJOG : an international journal of obstetrics and gynaecology. 2001;108(1):16–22.

49. Horowitz JA, Bell M, Trybulski J, Munro BH, Moser D, Hartz SA, et al. Promoting responsiveness between mothers with depressive symptoms and their infants. Journal of Nursing Scholarship. 2001;33(4):323–30.

50. Howell EA, Balbierz A, Wang J, Parides M, Zlotnick C, Leventhal H. Reducing postpartum depressive symptoms among black and Latina mothers: a randomized controlled trial. Obstetrics and gynecology. 2012;119(5):942–9.

51. Hui A, Back L, Ludwig S, Gardiner P, Sevenhuysen G, Dean H, et al. Lifestyle intervention on diet and exercise reduced excessive gestational weight gain in pregnant women under a randomised controlled trial. BJOG: an international journal of obstetrics and gynaecology. 2012;119(1):70–7.

52. Ingram L, MacArthur C, Khan K, Deeks JJ, Jolly K. Effect of antenatal peer support on breastfeeding initiation: a systematic review. CMAJ: Canadian Medical Association journal = journal de l’Association medicale canadienne. 2010;182(16):1739–46.

53. Janssen A, Desmarais L. Women’s experience with early labour management at home vs. in hospital: A randomised controlled trial. Midwifery. 2013;29(3):190–5.

54. Johnson Z, Molloy B, Scallan E, Fitzpatrick P, Rooney B, Keegan T, et al. Community Mothers Programme--seven year follow-up of a randomized controlled trial of non-professional intervention in parenting. Journal of public health medicine. 2000;22(3):337–42.

55. Jolly K, Ingram L, Freemantle N, Khan K, Chambers J, Hamburger R, et al. Effect of a peer support service on breast-feeding continuation in the UK: a randomised controlled trial. Midwifery. 2012;28(6):740–5.

56. Jolly K, Ingram L, Khan KS, Deeks JJ, Freemantle N, MacArthur C. Systematic review of peer support for breastfeeding continuation: metaregression analysis of the effect of setting, intensity, and timing. BMJ (Clinical research ed). 2012;344:d8287.

57. Karanja N, Lutz T, Ritenbaugh C, Maupome G, Jones J, Becker T, et al. The TOTS community intervention to prevent overweight in American Indian toddlers beginning at birth: a feasibility and efficacy study. Journal of community health. 2010;35(6):667–75.

58. Katz KS, Jarrett MH, El-Mohandes AA, Schneider S, McNeely-Johnson D, Kiely M. Effectiveness of a combined home visiting and group intervention for low income African American mothers: the pride in parenting program. Maternal and child health journal. 2011;15 Suppl 1:S75-84.

59. Katz KS, Rodan M, Milligan R, Tan S, Courtney L, Gantz M, et al. Efficacy of a randomized cell phone-based counseling intervention in postponing subsequent pregnancy among teen mothers. Maternal and child health journal. 2011;15 Suppl 1:S42-53.

60. Kaunonen M, Hannula L, Tarkka MT. A systematic review of peer support interventions for breastfeeding. Journal of clinical nursing. 2012;21(13–14):1943–54.

61. Kearney MH, York R, Deatrick JA. Effects of home visits to vulnerable young families. Journal of Nursing Scholarship. 2000;32(4):369–77.

62. Kenyon S, Jolly K, Hemming K, Ingram L, Gale N, Dann SA, et al. Evaluation of Lay Support in Pregnant women with Social risk (ELSIPS): a randomised controlled trial. BMC pregnancy and childbirth. 2012;12:11.

63. Kieffer EC, Caldwell CH, Welmerink DB, Welch KB, Sinco BR, Guzman JR. Effect of the healthy MOMs lifestyle intervention on reducing depressive symptoms among pregnant Latinas. American journal of community psychology. 2013;51(1–2):76–89.

64. Koniak-Griffin D, Anderson NL, Verzemnieks I, Brecht ML. A public health nursing early intervention program for adolescent mothers: outcomes from pregnancy through 6 weeks postpartum. Nursing research. 2000;49(3):130–8.

65. Koniak-Griffin D, Verzemnieks IL, Anderson NLR, Brecht M, Lesser J, Kim S, et al. Nurse visitation for adolescent mothers: two-year infant health and maternal outcomes. Nursing Research. 2003;52(2):127–37.

66. Kools EJ, Thijs C, Kester AD, van den Brandt PA, de Vries H. A breast-feeding promotion and support program a randomized trial in The Netherlands. Preventive medicine. 2005;40(1):60–70.

67. Kozinszky Z, Dudas RB, Devosa I, Csatordai S, Toth E, Szabo D, et al. Can a brief antepartum preventive group intervention help reduce postpartum depressive symptomatology? Psychotherapy and psychosomatics. 2012;81(2):98–107.

68. Kronborg H, Vaeth M, Olsen J, Iversen L, Harder I. Effect of early postnatal breastfeeding support: a cluster-randomized community based trial. Acta paediatrica (Oslo, Norway: 1992). 2007;96(7):1064–70.

69. Lee E, Mitchell-Herzfeld SD, Lowenfels AA, Greene R, Dorabawila V, DuMont KA. Reducing low birth weight through home visitation: a randomized controlled trial. American journal of preventive medicine. 2009;36(2):154–60.

70. Lee K. Intervention effects on maternal concepts of development for children’s cognitive outcomes. Journal of Human Behavior in the Social Environment. 2005;11(2):77–96.

71. Leis JA, Mendelson T, Tandon SD, Perry DF. A systematic review of home-based interventions to prevent and treat postpartum depression. Archives of women’s mental health. 2009;12(1):3–13.

72. Letourneau NL, Stewart MJ, Barnfather AK. Adolescent mothers: support needs, resources, and support-education interventions. The Journal of adolescent health : official publication of the Society for Adolescent Medicine. 2004;35(6):509–25.

73. Lewin S, Munabi-Babigumira S, Glenton C, Daniels K, Bosch-Capblanch X, van Wyk BE, et al. Lay health workers in primary and community health care for maternal and child health and the management of infectious diseases. Cochrane database of systematic reviews (Online). 2010(3):CD004015.

74. Lieu TA, Braveman PA, Escobar GJ, Fischer AF, Jensvold NG, Capra AM. A randomized comparison of home and clinic follow-up visits after early postpartum hospital discharge. Pediatrics. 2000;105(5):1058–65.

75. McCurdy K. Can home visitation enhance maternal social support? American journal of community psychology. 2001;29(1):97–112.

76. McDonald SJ, Henderson JJ, Faulkner S, Evans SF, Hagan R. Effect of an extended midwifery postnatal support programme on the duration of breast feeding: a randomised controlled trial. Midwifery. 2010;26(1):88–100.

77. McInnes RJ, Chambers JA. Supporting breastfeeding mothers: qualitative synthesis. Journal of advanced nursing. 2008;62(4):407–27.

78. McIntyre HD, Peacock A, Miller YD, Koh D, Marshall AL. Pilot study of an individualised early postpartum intervention to increase physical activity in women with previous gestational diabetes. International journal of endocrinology. 2012;2012:892019.

79. McKeever P, Stevens B, Miller KL, MacDonell JW, Gibbins S, Guerriere D, et al. Home versus hospital breastfeeding support for newborns: a randomized controlled trial. Birth (Berkeley, Calif). 2002;29(4):258–65.

80. McNaughton DB. Nurse home visits to maternal-child clients: a review of intervention research. Public health nursing (Boston, Mass). 2004;21(3):207–19.

81. Meghea CI, Li B, Zhu Q, Raffo JE, Lindsay JK, Moore JS, et al. Infant health effects of a nurse-community health worker home visitation programme: a randomized controlled trial. Child: care, health and development. 2013;39(1):27–35.

82. Milgrom J, Schembri C, Ericksen J, Ross J, Gemmill AW. Towards parenthood: an antenatal intervention to reduce depression, anxiety and parenting difficulties. Journal of affective disorders. 2011;130(3):385–94.

83. Morrell CJ, Spiby H, Stewart P, Walters S, Morgan A. Costs and benefits of community postnatal support workers: a randomised controlled trial. Health technology assessment (Winchester, England). 2000;4(6):1–100.

84. Morse C, Durkin S, Buist A, Milgrom J. Improving the postnatal outcomes of new mothers. Journal of advanced nursing. 2004;45(5):465–74.

85. Muirhead PE, Butcher G, Rankin J, Munley A. The effect of a programme of organised and supervised peer support on the initiation and duration of breastfeeding: a randomised trial. The British journal of general practice: the journal of the Royal College of General Practitioners. 2006;56(524):191–7.

86. Mulcahy R, Reay RE, Wilkinson RB, Owen C. A randomised control trial for the effectiveness of group Interpersonal Psychotherapy for postnatal depression. Archives of women’s mental health. 2010;13(2):125–39.

87. Mullany B, Barlow A, Neault N, Billy T, Jones T, Tortice I, et al. The Family Spirit trial for American Indian teen mothers and their children: CBPR rationale, design, methods and baseline characteristics. Prevention science: the official journal of the Society for Prevention Research. 2012;13(5):504–18.

88. Murphy CA, Cupples ME, Percy A, Halliday HL, Stewart MC. Peer-mentoring for first-time mothers from areas of socio-economic disadvantage: a qualitative study within a randomised controlled trial. BMC health services research. 2008;8:46.

89. Norr KF, Crittenden KS, Lehrer EL, Reyes O, Boyd CB, Nacion KW, et al. Maternal and infant outcomes at one year for a nurse-health advocate home visiting program serving African Americans and Mexican Americans. Public Health Nursing. 2003;20(3):190–204.

90. Olds DL, Kitzman H, Cole R, Robinson J, Sidora K, Luckey DW, et al. Effects of nurse home-visiting on maternal life course and child development: age 6 follow-up results of a randomized trial. Pediatrics. 2004;114(6):1550–60.

91. Olds DL, Robinson J, O’Brien R, Luckey DW, Pettitt LM, Henderson CR, Jr., et al. Home visiting by paraprofessionals and by nurses: a randomized, controlled trial. Pediatrics. 2002;110(3):486–97.

92. Olds DL, Robinson J, Pettitt L, Luckey DW, Holmberg J, Ng RK, et al. Effects of home visits by paraprofessionals and by nurses: age 4 follow-up results of a randomized trial. Pediatrics. 2004;114(6):1560–8.

93. Ostbye T, Krause KM, Lovelady CA, Morey MC, Bastian LA, Peterson BL, et al. Active Mothers Postpartum: a randomized controlled weight-loss intervention trial. American journal of preventive medicine. 2009;37(3):173–80.

94. Petrou S, Boulvain M, Simon J, Maricot P, Borst F, Perneger T, et al. Home-based care after a shortened hospital stay versus hospital-based care postpartum: an economic evaluation. BJOG : an international journal of obstetrics and gynaecology. 2004;111(8):800–6.

95. Polanska K, Hanke W, Sobala W, Broszkiewicz M. Smoking cessation intervention during pregnancy in a Polish urban community - what is the target population? Tobacco induced diseases. 2002;1(2):121–8.

96. Pugh LC, Milligan RA, Frick KD, Spatz D, Bronner Y. Breastfeeding duration, costs, and benefits of a support program for low-income breastfeeding women. Birth (Berkeley, Calif). 2002;29(2):95–100.

97. Ray KL, Hodnett ED. Caregiver support for postpartum depression. Cochrane database of systematic reviews (Online). 2001(3):CD000946.

98. Reid M, Glazener C, Murray GD, Taylor GS. A two-centred pragmatic randomised controlled trial of two interventions of postnatal support. BJOG: an international journal of obstetrics and gynaecology. 2002;109(10):1164–70.

99. Renfrew MJ, McCormick FM, Wade A, Quinn B, Dowswell T. Support for healthy breastfeeding mothers with healthy term babies. Cochrane database of systematic reviews (Online). 2012;5:CD001141.

100. Rohr F, Munier A, Sullivan D, Bailey I, Gennaccaro M, Levy H, et al. The Resource Mothers Study of Maternal Phenylketonuria: preliminary findings. Journal of inherited metabolic disease. 2004;27(2):145–55.

101. Roman LA, Gardiner JC, Lindsay JK, Moore JS, Luo Z, Baer LJ, et al. Alleviating perinatal depressive symptoms and stress: a nurse-community health worker randomized trial. Archives of women’s mental health. 2009;12(6):379–91.

102. Schmied V, Beake S, Sheehan A, McCourt C, Dykes F. Women’s perceptions and experiences of breastfeeding support: a metasynthesis. Birth (Berkeley, Calif). 2011;38(1):49–60.

103. Schuler ME, Nair P, Black MM. Ongoing maternal drug use, parenting attitudes, and a home intervention: effects on mother-child interaction at 18 months. Journal of developmental and behavioral pediatrics: JDBP. 2002;23(2):87–94.

104. Schuler ME, Nair P, Black MM, Kettinger L. Mother-infant interaction: effects of a home intervention and ongoing maternal drug use. Journal of clinical child psychology. 2000;29(3):424–31.

105. Sharp DJ, Chew-Graham C, Tylee A, Lewis G, Howard L, Anderson I, et al. A pragmatic randomised controlled trial to compare antidepressants with a community-based psychosocial intervention for the treatment of women with postnatal depression: the RESPOND trial. Health technology assessment (Winchester, England). 2010;14(43):iii-iv, ix.

106. Shaw E, Levitt C, Wong S, Kaczorowski J. Systematic review of the literature on postpartum care: effectiveness of postpartum support to improve maternal parenting, mental health, quality of life, and physical health. Birth (Berkeley, Calif). 2006;33(3):210–20.

107. Spiby H, McCormick F, Wallace L, Renfrew MJ, D’Souza L, Dyson L. A systematic review of education and evidence-based practice interventions with health professionals and breast feeding counsellors on duration of breast feeding. Midwifery. 2009;25(1):50–61.

108. Steel O’Connor KO, Mowat DL, Scott HM, Carr PA, Dorland JL, Young Tai KF. A randomized trial of two public health nurse follow-up programs after early obstetrical discharge: an examination of breastfeeding rates, maternal confidence and utilization and costs of health services. Canadian journal of public health Revue canadienne de sante publique. 2003;94(2):98–103.

109. Taft AJ, Small R, Hegarty KL, Watson LF, Gold L, Lumley JA. Mothers’ AdvocateS In the Community (MOSAIC)--non-professional mentor support to reduce intimate partner violence and depression in mothers: a cluster randomised trial in primary care. BMC public health. 2011;11:178.

110. Tappin DM, Lumsden MA, Gilmour WH, Crawford F, McIntyre D, Stone DH, et al. Randomised controlled trial of home based motivational interviewing by midwives to help pregnant smokers quit or cut down. BMJ (Clinical research ed). 2005;331(7513):373–7.

111. Tappin DM, Lumsden MA, McIntyre D, McKay C, Gilmour WH, Webber R, et al. A pilot study to establish a randomized trial methodology to test the efficacy of a behavioural intervention. Health education research. 2000;15(4):491–502.

112. Volpe EM, Bear M. Enhancing breastfeeding initiation in adolescent mothers through the Breastfeeding Educated and Supported Teen (BEST) Club. Journal of human lactation : official journal of International Lactation Consultant Association. 2000;16(3):196–200.

113. Wan MW, Sharp DJ, Howard LM, Abel KM. Attitudes and adjustment to the parental role in mothers following treatment for postnatal depression. Journal of affective disorders. 2011;131(1–3):284–92.

114. Watt RG, Tull KI, Hardy R, Wiggins M, Kelly Y, Molloy B, et al. Effectiveness of a social support intervention on infant feeding practices: randomised controlled trial. Journal of epidemiology and community health. 2009;63(2):156–62.

115. Weis KL, Ryan TW. Mentors offering maternal support: a support intervention for military mothers. Journal of obstetric, gynecologic, and neonatal nursing: JOGNN/NAACOG. 2012;41(2):303–14.

116. Wiggins M, Oakley A, Roberts I, Turner H, Rajan L, Austerberry H, et al. The Social Support and Family Health Study: a randomised controlled trial and economic evaluation of two alternative forms of postnatal support for mothers living in disadvantaged inner-city areas. Health technology assessment (Winchester, England). 2004;8(32):iii, ix-x, 1.

117. Wiggins M, Oakley A, Roberts I, Turner H, Rajan L, Austerberry H, et al. Postnatal support for mothers living in disadvantaged inner city areas: a randomised controlled trial. Journal of Epidemiology & Community Health. 2005;59(4):288–96.

118. Wiltheiss GA, Lovelady CA, West DG, Brouwer RJ, Krause KM, Ostbye T. Diet quality and weight change among overweight and obese postpartum women enrolled in a behavioral intervention program. Journal of the Academy of Nutrition and Dietetics. 2013;113(1):54–62.

119. Zelkowitz P, Feeley N, Shrier I, Stremler R, Westreich R, Dunkley D, et al. The cues and care randomized controlled trial of a neonatal intensive care unit intervention: effects on maternal psychological distress and mother-infant interaction. Journal of developmental and behavioral pediatrics : JDBP. 2011;32(8):591–9.

## Abbreviations

AB: Abstract
CINAHL: Cumulative Index to Nursing and Allied Health Literature
HIC: High income countries
LMIC: Low and middle income countries
MASCOT: Multilateral Association for Studying health inequalities and enhancing north–south and south-south Cooperation
OECD: Organisation for economic co-operation and development
PROGRESS-Plus: Place of residence, race/ethnicity, occupation, gender, religion, education, socioeconomic status, and social capital, and plus represents additional categories such as age, disability, and sexual orientation
RCT: Randomised control trials
SR: Systematic reviews
TI: Title
WHO: World Health Organization
